# Collaborating in a penta-helix structure within a community based participatory research programme: ‘*Wrestling with hierarchies and getting caught in isolated downpipes*’

**DOI:** 10.1186/s13690-021-00544-0

**Published:** 2021-03-06

**Authors:** Katarina Sjögren Forss, Anders Kottorp, Margareta Rämgård

**Affiliations:** Department of Care Science, Malmö University Faculty of Health and Society, 205 06 Malmö, Sweden

**Keywords:** CBPR, Collaboration, Health promotion, Penta-helix

## Abstract

**Background:**

In the light of the existence of social inequalities in health, a CBPR (Community Based Participatory Research) programme for health promotion started in Malmö, Sweden, in 2017. The programme was based on a penta-helix structure and involved a strategic steering group with representatives from academia, voluntary organisations, the business sector, the public sector, and citizens from the community where the programme took place. The aim of this study was to explore how the penta-helix collaboration worked from the perspectives of all partners, including the citizens.

**Methods:**

Individual interviews, that were based on a guide for self-reflection and evaluation of CBPR partnerships, were conducted with the representatives (*N* = 13) on three occasions, during the period 2017–2019. A qualitative content analysis was used to analyse the interviews.

**Results:**

Six themes emerged from the analysis, including Challenges for the partners in the penta-helix collaboration; Challenges for the professionals at the local level; Citizen-driven processes are important for the penta-helix collaboration; Health promoters are essential to build trust between different sectors of society; Shift of power; and System changes take time. The analysis shows that the penta-helix collaboration worked well at the local level in a governance-related model for penta-helix cooperation. In the overall cooperation it was the citizen-driven processes that made the programme work. However, the findings also indicated an inflexibility in organisations with hierarchical structures that created barriers for citizen involvement in the penta-helix collaboration.

**Conclusion:**

The main issue uncovered in this study is the problem of vertically organised institutions where discovery and innovation processes flow down from the top, thereby eliminating the essential input of the people and community that they are supposed to serve. The success of the programme was based on an interprofessional cooperation at a local level, where local professions worked together with voluntary organisations, social workers, CBPR researchers from the university, citizens and local health promoters.

## Background

The existence of social inequalities in health is well established in the world. To reduce these inequalities, it is essential to take action on the social determinants of health in a broader perspective and improve the conditions in which people live and work [1]. Health inequalities can be reduced through increased social justice, achieved by supporting the development of both individual and collective engagements. One way to do this is through community-based empowerment initiatives. Engaged and empowered communities can then provide supportive environments and create positive social norms that facilitate individuals to gain motivation, confidence and self-management skills [2]. Furthermore, to create more equal health in socially vulnerable areas there is also a need for new strategies. A multisectoral approach (MSA), involving various stakeholders from different sectors in the society, is important for solving complex societal challenges such as inequalities in health [3]. The challenge in such collaboration is to engage the stakeholders to work with intersectoral actions in order to create sustainability solutions. The World Health Organization (WHO) uses the term intersectoral actions as an umbrella term to highlight actions in a collaboration undertaken by sectors outside the health sector. The health sector is just one partner working within the MSA partnership, as a wide range of social and environmental factors affect health and wellbeing [ibid.]. In this paper, we argue that the community needs to be a more active partner in such multisectoral collaboration to create sustainability solutions in socially vulnerably areas.

The health promotion programme that will be presented in this paper was undertaken by the university and focused on stimulating MSA work outside the healthcare sector, in a close cooperation with stakeholders from different sectors and the citizens in the community.

**Fig. 1:**
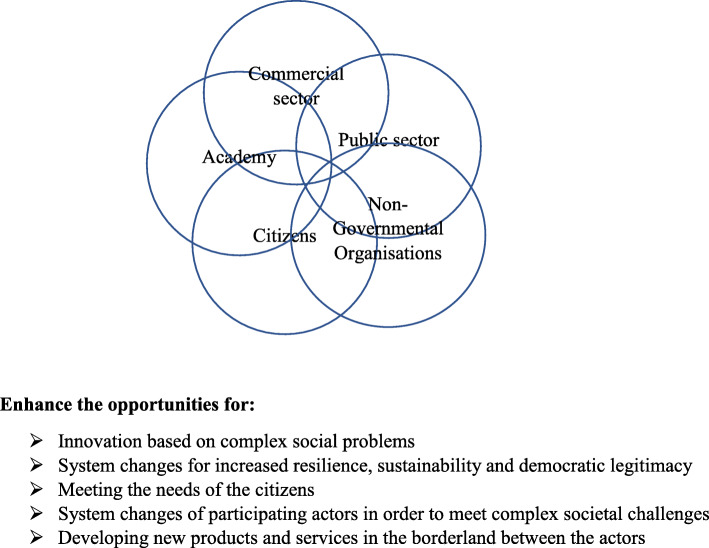
Penta helix structure for the programme Collaborative Innovations for Health Promotion, Malmö Sweden

A penta-helix model (Fig. 1) for collaboration has been developed for the programme, where citizens from the community work with stakeholders and academia in building a partnership driven by the community. In parallel with building up a local collaboration for health promotion, the partners engage in policy-changing processes on a structural level to remove barriers for governance collaboration.

### Collaboration in health promotion

Several factors, both internal and external, affect the health of individuals. Not all of these are directly under the control of the individual, for example, the state of the environment, the community and the social context. Thus, the health of individuals must be viewed as not only an individual but also a common responsibility, which means that many sectors must be involved and collaborate in this work, not only the healthcare sector [4]. Consequently, a holistic approach, with intersectoral action and partnership, is a cornerstone in promoting health and health equity [5], as it includes partnerships across the spectrum of health promotion initiatives locally, regionally and nationally [6]. In order to be successful, it is therefore important to identify opportunities for collaboration, negotiate agendas and promote synergies [4]. By working in an effective partnership, partners can achieve better results than by working on their own and they can also produce synergy effects, as their different skills, resources and knowledge complement each other and result in more effective solutions [7]. However, it is vital that the implementation of a cross-sectoral partnership builds on what is already known about effective collaboration processes [8]. A review assessing the impact of alliances of partnerships for health promotion, concluded that intersectoral collaborations to promote health between public, private and non-government sectors (i.e., non-government organisations), do work [9]. Also, the more local community partners are involved in setting the agenda for actions as well as in the implementation of health promotion strategies, the greater the impact of the partnership. Activities organised by volunteers also ensure the optimal advantage from a community approach (ibid.). Worldwide, there is no commonly accepted theory of health promotion partnership, but numerous theoretical frameworks exist [8]. The health promotion programme that will be presented in this paper, was built on CBPR (Community Based Participatory Research).

CBPR focuses on addressing determinants of health from both a social and an environmental perspective through active engagement of the community members and other involved actors throughout the research process. In CBPR, systematic efforts are made to integrate the community in decision-making processes and encourage active participation in those processes by creating a common understanding of local phenomena as well as practices specific to the community in question. This will contribute to the development of different innovative strategies to promote social changes [10].

One of the characteristics of CBPR that distinguish it from other participatory methods is the equal partnership at all stages of the research process and the importance of each partner defining their roles clearly in the beginning of the project. Thus, it is, as expressed by Holkup et al., ‘an appealing model for research with vulnerable populations’ [11]. Wallerstein et al. [12] argue that CBPR actually extends beyond ‘shared leadership’, to ‘community-driven’ approaches, where communities decide research priorities, participate in all research steps, and strategize to co-implement actions from findings. They define CBPR as ‘collaborative efforts among community, academic, and other stakeholders who gather and use research and data to build on the strengths and priorities of the community for multilevel strategies to improve health and social equity’ [ibid.]. Participatory research is based on theories by Lewin [13], who was oriented towards changing organisations and bridging the gap between theory and practice in working with different stakeholders. CBPR researchers have also been focusing on social movements, often from a perspective adopted from Freire [14] and aiming at fostering democracy, participation, social transformation and empowerment among citizens [12]. Empowerment can be viewed from two levels, the individual level and the community level [12]. At the individual level, empowerment is about the general capacity of an individual to feel strong enough to make choices in their lives, while empowerment at the community level refers to the capacity of a community, through the participation process, to reach collectively defined goals [ibid.]. Involvement at both individual and community levels is required for the process to be successful [15]. However, crucial for empowering a whole community is that the community partners also have developed more power over the place and that they feel attached to the material environment. Wallerstein and colleagues [16] have developed a conceptual model for working with CBPR partnerships. The model involves four dimensions: context, partnership processes, intervention/research and outcomes. In this paper, we focus on the processes and outcomes of the partnership. The partnerships and the PAR (Participatory Action Research) interventions in the CBPR programme have been published in a previous study [17].

### Developing a programme with CBPR

In 2017, a CBPR programme, Collaborative Innovations for Health Promotion, was established in the city of Malmö, Sweden, to create new ways to improve health, primarily through participatory and collaborative penta-helix strategies from a community perspective in a new health promotion platform. The overall aims of the programme were to develop and study health-promoting activities based on participatory research methods.

The programme for health-promoting collaboration is built on Freire’s empowerment processes [14] as well as on Lewin’s [13] goal to change power relations inside organisations, and the above-mentioned conceptual model was used for planning the programme. The planning started with pre-activities to frame the community context and identified the partnership practice and participatory decision-making around interventions, such as involving citizens from the community in all phases of the research.

The programme took place in Malmö, at the initiative of Malmö University and the community, with funding from Vinnova (Sweden’s innovation agency) to the sum of SEK 8.6 m. Malmö is the third biggest city in Sweden, and the programme was developed in a geographical part of Malmö, Lindängen, with about 7600 citizens, approximately 75% of whom are first- and second-generation migrants [13]. Unemployment is about 50% in both men and women in the area [18], which can be described as relatively deprived, with high criminality and considerable inequalities in health.

**Fig. 2:**
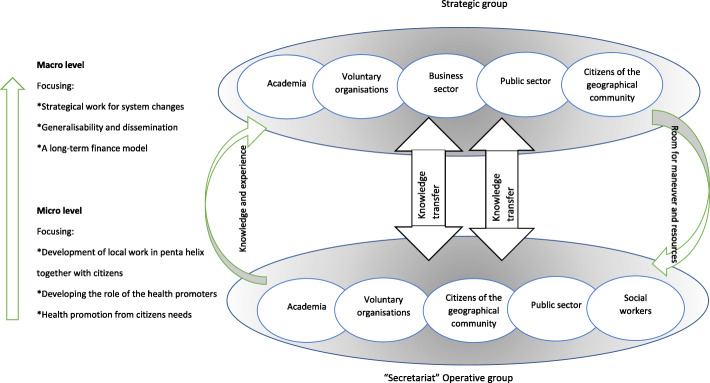
Project organisation for the programme. Collaborative Innovations for Health Promotion, Malmö, Sweden

A penta-helix structure characterized the programme, which involved the following actors: Academia, the public sector/the state, the business sector, non-governmental organisations, and partners from the community (Fig. 1). In the penta-helix structure, all actors collaborate and are engaged at the local, operational, and strategic levels and in this programme, the partners worked together in a partnership based on the CBPR principles (Fig. 2).

A strategic steering group was formed at an initial stage in the CBPR planning by organisations that showed an interest in and had a specific focus on promoting health in deprived city areas. This included representatives from academia (Malmö University), voluntary organisations (The Red Cross, Save the Children, Skåne Sport Federation), the business sector (pharmacy, housing company, technology company, oral health company), and the public sector (Region of Scania, Malmö City, Finsam – a state financial coordination covenant in rehabilitation). Furthermore, citizens of the community were represented in the steering group.

The steering group, whose cooperation was multisectoral, that had an MSA cooperation, worked based on CBPR principles [19], taking the shared identity of the community into account and basing the programme on the strength of community collaboration. The goal was also to make the programme a long-term commitment in the community and for knowledge from the programme to result in actions for policy changes within the partners’ own organisations so that they were able to distribute the programme. The steering group also had an advisory role. The programme was built on vertical and horizontal collaboration. Horizontal cooperation refers to a voluntary and a more relational and interpersonal cooperation between different actors working in a more governance-directed way, while vertical cooperation takes place in a strong hierarchy [20]. Horizontal organisations have a greater need for legitimacy compared to vertical ones, for example, through delegated management decisions. Vertical organisations need to report to their management in order for a project to be given legitimacy (ibid.). In order to be able to change policies, the programme has to function in both vertically and horizontally driven organisations (Fig. 2).

On an operative level, there was a local organisation, a secretariat, working in a penta-helix structure with professionals from different sectors employed by partners from the steering group (i.e., social workers from Malmö City, professionals from the Red Cross and the municipality, and PAR researchers from the academy). The secretariat could be seen as the hub in the programme, working locally in the community, close to the citizens and with the mission to create dialogues, build trust and mobilize the citizens in an interprofessional and intersectoral cooperation (Fig. 2). In the secretariat, professionals and researchers from health and social care worked together with voluntary organisations and *local* health promoters (henceforth called health promoters).

The health promoters (*N* = 6) were citizens in the community that had expressed their interest in the programme during the ‘future workshops’ organised before building the partnerships. They had been chosen by the citizens to represent the community in the CBPR planning and could be seen as facilitators for participant recruitment and language interpretation, as well as building relations and trust. They had continuous dialogues with the citizens [21], whom they represented, and they were also engaged in the steering group. They participated actively in creating the CBPR model of the research platform. The health promoters were all first-generation migrants (from, for example, Europe and the Middle East), differed in gender and age, and represented diverse ethnic groups. They had lived in Sweden for many years, spoke Swedish, and had earlier been involved in different activities in the community.

### Pre-programme activities

Before the programme started, the project leader (MR) did some field work to explore existing activities in the community and there were already some ongoing activities to build on. Two of the voluntary organisations, the Red Cross and Save the Children International, had different activities in the community and they had, thus, already started the process of building trust among the citizens. Furthermore, the public sector had previously organised activities in the community, also with the ambition to work multisectorally, but those activities were governed by Malmö City, as opposed to the equal partnership in the CBPR model used in the programme in this study. The professionals worked in their own organisations and there was no organised work between the different professionals regarding health-promotion work. The work had been in the shape of organised field work and the field workers had established contacts in the community as well as some urban meeting places. Some of these social meeting places still existed when the programme started, and there were also some field workers left in the community. However, the number of field workers was substantially reduced as a consequence of financial cuts, and these changes had resulted in some mistrust among the citizens. In the social meeting places in the area, some participation from the citizens could be observed; it was not, however, driven by the citizens themselves, and consequently their scope of action was confined. Moreover, knowledge about the citizens’ health from their own perspective, and based on what they themselves perceived to be their needs, was lacking, and there was also a mistrust of the healthcare system among the citizens.

### Creating CBPR principles for social change and empowerment

Before starting the planning and the collaboration process, ‘future workshops’ [22] were organised. Citizens of the community were invited to those workshops in order to identify community strengths and to customize the programme to the citizens’ own perceived needs regarding their own health promotion. A total of 150 citizens (adults and children) participated in the workshops. The health needs identified by the citizens were: lack of physical activity, poor oral health and mental health problems. The citizens also wanted to create a sense of place and learning in co-creative so-called living labs for health promotion in social meeting places in the area. Together with all partners at the local level, they created a CBPR model, inspired by Wallerstein et al. [12], for planning these labs, suggesting interventions and confirming the collaboration, in order to set goals for the organisation and an overall programme. In total, over 320 citizens, of different ages, cultural backgrounds and gender, participated in the six labs every week.

By using the penta-helix structure both in a vertical (steering group) and a horizontal (local secretariat) integration [20], in which the citizens had just as big an impact as other actors, and by involving them in the whole process of constructing the programme, a stronger community-driven approach could be created. Furthermore, processes of empowerment, another central component of the programme, could be initiated.

When facing a global challenge such as health equity, the involvement of the whole society is required, as well as a collaboration between different sectors, as in this community-driven MSA. In order to reduce inequities, the programme therefore used a penta-helix perspective that also empowered the citizens by making them equal partners. The citizens here played a strategic role, since they transferred the knowledge from the community directly to the strategic level for policy changes. The health promoters, recruited among the citizens from the area, were employed by the platform and formed part of the local secretariat working with partners from different sectors, and they were not committed to the culture and norms of a certain organisation. However, in CBPR projects, it is important to understand how the collaboration between the partners develops during the process, in order to gain knowledge about what societal effects and social impacts can be achieved through such cooperation. Thus, a process evaluation is important in order to understand how the collaborations have developed during the programme. The aim of this study was to explore how the penta-helix collaboration worked from the perspectives of all partners, including the citizens.

## Method

The partners of the steering group (*N* = 13) and the secretariat (*N* = 8), as well as members of the community (*N* = 3), were interviewed on three occasions, in late 2017, in October 2018, and in May 2019. The interviews conducted in 2017 were performed by a project research assistant, and the second and third interviews were all performed by the first author (KSF).

The semi-structured interviews were based on a guide for self-reflection and evaluation of CBPR partnerships constructed by Wallerstein [23], ‘*Partnership Interview Guide; Qualitative Study Instrument, 2011 [adapted 2015]*’, and it consisted of questions from seven different perspectives with focus on the partnership: *involvement*; *context*; *partnership and group behaviour*; *intervention and design*; *intervention and policy impact*; *sustainability*; and *advice to others*. The same guide was used during all three interviews. In the interviews, the partners represented their organisation and not themselves. The interviews took place at the partners’ workplaces and citizens’ meeting places and lasted between 30 and 60 min, and they were tape-recorded and transcribed before the analysis began. Before the interviews, the participants were given oral and written information about the study and were asked to sign an informed consent form about the study and their involvement in the research. The participants were ensured confidentiality and they were also informed that all results would be presented at a group level so that no individual voices would be identifiable. Consequently, all quotations in the study are anonymous. Data were stored securely and anonymously in compliance with the Data Protection Act. No participant declined participation.

An inductive qualitative content analysis as described by Elo and Kyngäs [24] was used to analyse the interviews. First, the transcripts were read individually numerous times to obtain an overall understanding of the data. Second, meaning units of the text that corresponded with the aim were condensed and coded manually using colouring pens. As a third step, the coloured codes were interpreted and compared, to find similarities and differences, and then (as a fourth step) they were sorted into tentative themes without losing their content (ibid.). The first and the last author took the lead in the initial part of the analysis; however, during the entire process, all authors separately read, analysed and discussed the text to enhance the best possible account of the meaning found in the text. Finally, and as the fifth step, the authors agreed on six themes.

## Results

The six themes – *Challenges for the partners in the penta-helix collaboration*; *Challenges for the professionals at the local level*; *Citizen-driven processes are important for the penta-helix collaboration; Health promoters are essential for building trust between different sectors of society; Shift of power;* and *System changes take time –* were identified and considered to reflect the perspectives of the partners.

### Challenges for the partners in the penta-helix collaboration

All partners experienced different challenges in the penta-helix collaboration. In the first part of this theme, the main challenges encountered by each of the different partners are described.

The partners from academia stated that the main challenge for the partnership was to find cross-border solutions in the collaboration that would persist over time. Partners from voluntary organisations highlighted problems for organisations that based much of their work on volunteers, i.e., work without pay. Also, the voluntary organisations get funding from different sources and often only for periods of one or two years, which makes it difficult to work in a long-term perspective. The challenge for the business sector was that they work in lean organisations without any extra resources for participating in meetings and making plans for initiatives lasting over time. As for the partners from the public sector, the challenge was to get their own organisations to understand the CBPR model and to find their role in the programme from a bottom-up perspective, in contrast to their usual top-down way of working. Finally, the challenge for the partners representing the citizens was to make the citizens understand that they had to be engaged in the community’s workshops to be able to get their ideas involved in the programme. One of them stated*:*

*The programme got ideas from the people, from the workshop, from women. Yes, if they (citizens) not come to the workshop, there’s nothing.*

At the start, some organisations, for example, the Red Cross and Save the Children, were more visible than other organisations that found it more challenging to find their role in the project and the penta-helix collaboration. Some partners from the public sector were afraid that there would be expectations from other partners that they would not be able to live up to after the end of the project. Even in a big organisation there might not be unrestricted economic resources, and funding is often earmarked for special purposes. One partner from the public sector said:

*When we engage in a partnership there might be expectations that we are a kind of bank that it’s good to discuss with ... like …* ‘*we have some difficulties with funding for the project … maybe you can ...’ … I sometimes feel that when we collaborate, people expect the money to come.*

Thus, to encourage their multisectoral work, many partners in the penta-helix collaboration wished to have more knowledge about each other’s organisations and their prerequisites for and contributions to the programme, as it was seen as essential in order to avoid both misunderstandings and unrealistic expectations.

Another challenge described was that of taking the programme to a long-term sustainable solution that was not based on external financing. During the third interview, many partners also emphasized the importance of creating a democratic structure in the community for the health promoters to build on, as the programme, from a sustainability perspective, cannot be their funding base in the future. Some of the partners representing the university, highlighted the importance of not seeing the programme as an objective study object, as this might risk losing the bottom-up perspective that is so important in CBPR research. In contrast, especially partners from the business sector stated that it was important not to forget that the programme was above all an innovation programme and not only a research programme, and one of them declared:

*The innovation should not be lost in favour of research.*

However, one of the biggest challenges described was the inflexibility and stiffness in the partners’ own organisations that resulted in their feeling governed and not having enough room for action in the steering group. This inflexibility could be related to the vertical structures of a government model with no tradition to work from a bottom-up perspective. One of the partners from the public sector described it like this:

*We need to rethink and build new local horizontal systems instead of the old, vertical systems (downpipes) that we are still working in.*

### Challenges for the professionals at the local level

Rigidity in their own organisations was also highlighted among the partners at the operational level as being one of the biggest challenges. In the programme, the partners were close to each other and the citizens in the community and much of their work was about building relations and trust between people and organisations in order to deal with the complex issues at hand. They often worked as concrete problem solvers, helping each other, and, thus, needed to be flexible and to make decisions and act quickly based on collaboration between organisations. However, they frequently encountered a lack of flexibility on the part of their own organisations with regard to being able to act together with others, something that one partner described like this:

*… wrestling with hierarchies and getting caught in isolated downpipes.*

Most of the organisations were not used to working from the bottom-up perspective, and at the operational level the challenge for the partners was both to find constructive solutions for ensuring the progress of the programme and to find a balance in relation to their own organisation. One partner at the operational level stated:

*Systems that were built and worked in the 1970s don’t work today, as society has changed a lot. Organisations need to change to meet the needs of society and they are at different levels in this development. While some have started, others haven’t yet realised that they do need to change.*

Mobilization of knowledge was above all seen at the operational level, where people from different organisations and backgrounds worked together close to the citizens. They felt that even if their knowledge base was sometimes different, what they had in common was the hierarchical structure in their own organisations that often complicated their work and consequently acted as a barrier in the programme.

### Citizen-driven processes are important for the penta-helix collaboration

At the operational level, the partners with their different backgrounds had a broad range of contacts, which was seen as a strength as well as necessary for the programme. The partners could make use of each other’s competencies in the CBPR research planning model, something which has become increasingly important during the programme. One of them stated:

*We see our work as a social change built on collective intelligence.*

The partners had different opinions about the use of different tools for CBPR, such as the model and how it could lead to policy changes and then result in an increased citizen influence. Some, above all partners from the university and the voluntary organisations, who already were familiar with the model, asserted that it would have been impossible to come that far with regard to building trust, without the model. One partner from the voluntary organisations stated:

*The CBPR model is a central part of the programme and that was an important prerequisite for us to be a part of the programme. It gives structure and makes the bottom-up perspective visible.*

**Fig. 3:**
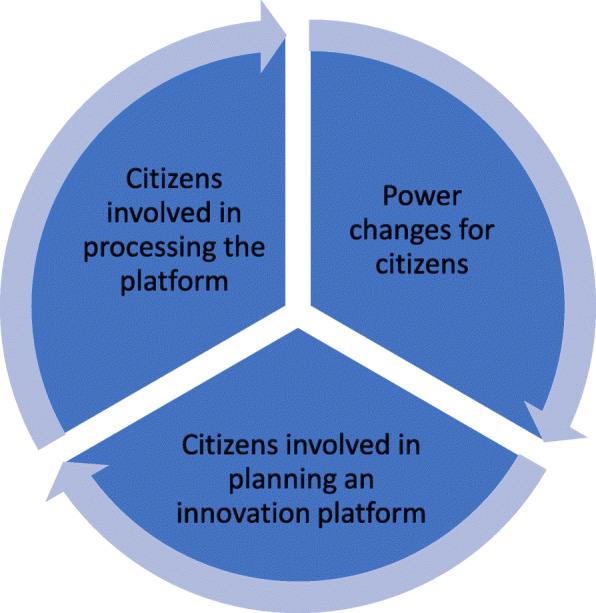
Circle of action for citizens from the programme Collaborative Innovations for Health Promotion, Malmö, Sweden

Others claimed that the CBPR model had worked well and had provided security and stability to the programme, as well as increased impact and participation. However, it was not the model itself but rather the citizen influence in all parts of the programme, with the ‘future workshops’, the CBPR planning, and the health promoters as well as the citizens being involved at all levels, at both the steering group and the operational level, that was the key factor of the programme’s success (Fig. 3). The people involved in the programme, especially the health promoters, and their commitment and building of confidence and relations, were more important for the programme than the model. If a programme such as this is not successful in building confidence and relations, it will fail regardless of using a model or not, many partners argue. The partners experienced that tools, for example, for planning the model, are needed, but it is citizen-driven processes that generate results, not a specific tool/model.

### Health promoters are essential for building trust between different sectors of society

All partners in the steering group, as well as in the secretariat, pointed out the importance of the health promoters, who had, to a large extent, contributed to the success of the programme, as they mobilized to build a sustainable society based on the citizens in the community. The health promoters created a physical link between the different sectors and, by doing so, facilitated communication and intersectoral actions between all partners. By also making sure that different organisations worked as little as possible in isolation to solve the complex problems addressed with/for the citizens, the health promotors facilitated a work process based on trust and collaboration between all partners in the penta-helix model. One partner representing one of the voluntary organisations said:

*In such an area, processes of building confidence are needed. The health promoters know their community and can build confidence to the residents. They are the link that is needed between the residents and the society to build confidence.*

However, as the role of the health promoters was new, it was perceived as both a great opportunity and a challenge. One partner, representing the university, described it like this:

*As the role of the health promoters is a new concept, it is also important to discuss how their role will be formalised without making it too formalised. Also, how can the health promoters be a part of and work in already existing systems, or do we need to find a new platform for their work?*

The health promoters worked across different sectors in the society. However, all partners described it as a challenge to know which sector should employ the health promoters in the future and how the financing of them should be solved when the programme was finished. Some of the health promoters worked voluntarily in the beginning of the programme, while others had an employment that was financed by different organisations. For the organisations involved in this work, above all the Red Cross and Save the Children, it was important that the different conditions (voluntary work/employment) were clear, in order to avoid a situation where people who do voluntary work for the organisations want to be remunerated.

### Shift of power

Above all, the partners from the university and the voluntary organisations stressed the importance of always being aware of the imbalance of power that might arise when people with different prerequisites work together. The majority of the partners in the steering group were used to being in such environments, representing different organisations, while the citizens of the community represented themselves as partners of the community and did not have earlier experiences of being in a steering group. It has been a vital process during the whole programme to make the partners representing the citizens visible and listen to them as important and equal partners, something which the majority of the partners, including the partners representing the citizens, felt had worked out well. When the partners representing the community were asked about power, they stated that during the programme they had seen that their role had become increasingly important and that they had made a difference. From their perspective, they defined this as power, and one of them said:

*If I explain to the people what you do in the projects and I come back to the steering group and explain what they want and I got a tip, perhaps can I help a lot in that way. Because I contact all the time the people and take their questions to the steering group and then bring the answers back. They trust me. It’s power.*

The partners at the operational level stated that they could see power changes at an individual as well as at a community level, changes that might be explained by the different phases of the programme. Those partners who worked at the operational level worked with health promoters, and with other local stakeholders, and had much more contact with the citizens in the community, so they could see actual changes and that the citizens had got more power over their space. The citizens were more enterprising and started up things on their own initiatives. They were now helping themselves more and more, and they asked the local stakeholders if they wanted something and suggested changes. The citizens were also increasingly aware of their rights. During the programme, the partners at the operational level had also seen a shift in power that one of them described like this:

*When the programme started, it was the organisations behind the programme that had the prerogative to interpret human rights, but now individuals have more and more started to own these rights.*

According to the interviews, the programme shows a shift in power relations, due to the fact that, in the steering group, citizens of the community were partners with persons working on a strategic level (such as a pro-vice-chancellor, and executive directors). For many of the partners in the steering group, this, together with the bottom-up perspective of the research, was a new way of working, and during the programme it has been obvious that they now all see the importance and value of working in this kind of partnership. One partner representing the university said:

*As citizens from the community are partners of the steering group, it is impossible to talk above their heads and, consequently, the perspective of the citizens is always in focus and the bottom-up perspective is not forgotten.*

The partners expressed the importance of pointing out the uniqueness of the programme, which differs from earlier research programmes in that the role of the citizens is equal to that of the other participants in the programme, as they are involved in the design of the programme. Moreover, all partners wanted to highlight the health promoters, who are also unique in this programme, in their work to involve the citizens in the community. This is how one of the health promoters described the work:

*I feel like an important person right now. The fact that everybody tells me …, I can tell to the steering group and everyone listens to my voice. Today I can, you know … gather the voices of other women and take to the steering group. Yes, and there’s someone who listens to me.*

The professionals at the operational level also highlighted changes related specifically to the women in the community; the project created more space for manoeuvre among the women and made them more visible in the community. From their work in the local place, the professionals could see that the women were ‘the hearts of their families’. They argue that strengthening the empowerment of the women at an individual level is central for achieving empowerment at a community level. The citizens themselves also saw the importance of focusing on the women, and one of them stated:

*I think women are up for it first, then in the family can everyone do together activities, or they will help each other. Do a lot, because the women are important people in the family.*

### System changes take time

All partners experienced different barriers in their own organisations, for example, hierarchies for decision-making. However, they all felt that their work and collaboration in the steering group worked well and they saw themselves as working in a network model of governance. All partners experienced a high participation and no hierarchies in the collaboration of the steering group. The composition of the group of partners, with regard to their experiences and competences, was described as good, and so was the trust and respect between the partners. Even if they did not always agree with each other, they highlighted the importance of everyone contributing with their different perspectives, mobilizing their knowledge and learning from each other.

For most of the partners in the steering group, working in a multidisciplinary way, according to a penta-helix structure, was a new experience and more complex and difficult than many of them had realised before the programme started. A top-down perspective, which does not build relations but instead demolishes them, could be seen among some of the partners, for example, in the attitude ‘what’s in the programme for us’, expressed like this by a partner from the public sector:

*The results from the programme can help us in our work in other communities with the same problems and challenges as we can see in this community.*

During the programme and over time, changes could be seen in how the programme was viewed by the partners of the steering group. This progress was noticeable when comparing the first interview to the second and third interviews, where the focus was changed to ‘what can we do for the programme?’ Furthermore, when interviewed the third time, it could be seen that some of those partners had started the process of changing their own inner structures, something which is, however, a long process. The partners pointed out that the programme is about impact and that changes take time, especially on a society level. When interviewed the two first times, the partners stated that even if it was possible to see positive changes on an individual level, it was too early to say anything about the benefits or results the programme might generate. Moreover, the programme was in too early a phase for them to say anything about changes at the policy or system level. In the last interview, however, some partners stated that the health promoters was now at a point where they started to make a difference at a policy level, which meant that the partners could show their organisations that the work of the health promoters had a considerable effect, and that this penta-helix collaboration was the new way to work for the future.

All partners asserted that the involvement of the university was positive and brought valuable aspects of research to the local level in the community, which strengthened the programme. This was of importance when reporting back to their own organisations to suggest policy changes. Further, all partners argued that the university inspires trust and represents secure ethical values that are necessary in this kind of programme, so that business or political interests do not take over. Partners from the voluntary organisations also said that one of the most important aspects of the programme was the research performed by the university, and the fact that they could present the results of that research to their managers at a national level. The voluntary organisations had experiences of working in a community-based way before; however, this was the first time research was so deeply involved, something that provided new dimensions and more value as compared to other projects.

The partners representing the citizens pointed out that thanks to the involvement of the university the citizens in the community had got a favourable view picture of the university, and one of them said:

*The path to the university has become shorter.*

## Discussion

This study was based on a programme that sought to create new ways to promote health, primarily through participatory and collaborative penta-helix strategies from a community perspective in a health promotion platform. The study aimed to explore how the penta-helix collaboration worked from the perspectives of all partners.

Our findings showed that the partners experienced the collaboration as challenging in different ways, as it was not easy or natural for all to work in a penta-helix collaboration. All partners were satisfied with the goal to work with community-driven solutions and the bottom-up perspective in an MSA. However, there were problems with intersectoral actions in vertical organisations. We could see both inflexibility and organisations with hierarchical structures that were not ready to work from a bottom-up perspective.

Moreover, all involved organisations had some problems. The inflexibility was above all seen on structural levels in the partners’ own organisations, however, and they felt that their MSA collaboration in the steering group worked well. The health promoters’ function as essential brokers between different sectors supported the intersectoral actions from a community perspective in and between the operational level and the steering group. Brokers are a well-known concept in many community health promotion programmes [25]. In this programme, the brokers were health promoters engaged in building the programme on all levels: in the strategic work in the steering groups, as well as in the secretariat and in the labs. This contributed to more community-driven empowerment processes in the MSA collaboration.

Most of the problems and frustration caused by the inflexibility were seen at the secretariat, where the partners worked close to the citizens and needed to be flexible in, for example, using each other’s resources, such as premises and staff. As a consequence, and thanks to their local collaboration in the penta-helix secretariat, and with the health promoters, they found new solutions and were often thinking outside the box, and all partners could see that their local collaboration facilitated a cross-sector network that increased the collaborative governance. As Svanholm et al. [26] argue, to make a health promotion programme sustainable over time, gathering different organisations in the same geographical area, could facilitate communications between stakeholders and the citizens. Empowerment brokers from the area strengthened the sustainability of this programme.

Wallerstein et al. [12] conclude that there is a need to plan for equal partnership in collaboration with the community in an iterative process that is community driven. Engaging communities in large ‘future workshops’ before starting the planning process with them was helpful in creating the MSA collaborations from the citizens’ perspective of health. Using the themes from those workshops in a CBPR planning, made the programme more community driven and enabled all partners to actively participate in creating the goal for the partnership and the actions in the labs.

In such a programme, it is important to keep the partnership open and see it as an ongoing process that is possible to influence during the whole programme, in order to avoid a decline in creativity. The way the partners described the decision process, decisions were developed during the course of the programme, and it could, thus, be seen as more of a governance networking structure for implementing the programme locally. The advantage of this could be a more dynamic decision process; the disadvantage, however, might be that the decision process is not always clear to everybody.

The challenge of the sustainability of the programme was also highlighted. However, this challenge is not unique to our programme and the sustainability of health promotion initiatives has frequently been discussed [27]. Sustainability often depends on the role of stakeholders outside of government and their capacity to create social networks or partnerships to sustain their efforts (ibid.). A newly presented review of factors for the sustainability of health promotion programmes showed the importance of a strong relationship between the involved partners as well as a participatory approach for achieving sustainability [28]. In this programme, the members of the secretariat stated that from a sustainability perspective, it would be better to work in a governance manner and shift the power from the steering group to the operational level, as their close collaboration with the citizens as well as with the health promoters was a cornerstone of sustainability.

This could be seen as a shift of power as well as an empowering process. According to Flynn et al. [29], professionals should work together with community members rather than directing them to create empowering participation, and Gillies [9] elucidated the importance of involving local community partners for getting the most out of the partnership. In the current study, the professionals in the secretariat, as well as the health promoters, can be seen as such partners. The professionals in the secretariat organised the local work together with the health promoters and the citizens, without involvement from the structural level. Thus, power was shifted from a structural to a local level. Consequently, the programme was driven by the citizens and their involvement, a shift of power that can be seen as the success of the programme.

From our findings it emerged that power was seen from different perspectives. Many of the partners in the steering group considered themselves to represent a structure with hierarchical elements of power and stated that the citizens represented in the steering group did not have such a structure to back them up, which resulted in an imbalance of power. Accordingly, this gave a direct focus on citizen influence, as it was impossible to overlook the citizens when they were represented, and had an active role, in the intersectoral actions and in building the programme. Laverack [30] asserts that individuals with power can use their own power to provide an environment to those without power, for example, marginalized groups. When empowerment is achieved, the marginalized groups are able to improve their conditions and sustain them over time. A powerful process of empowerment could be seen during the programme among the health promoters, who grew in their role and grew together, in a process resulting in their believing in themselves and in the citizens in the community. Together they developed a spirit characterized by the attitude that ‘together we can change things and make a difference’. The programme consequently helped to empower the citizens by involving them as equal partners in the penta-helix structure.

In the system, there is an inertia that can be explained by the hierarchical structures in a governmental model that hinder the structural level from working in a penta-helix perspective, involving citizens as equal partners in cooperation. As Wallerstein et al. [12]) conclude, if power relations are changed, the management system and the structure of the organisations must also be changed at a policy level, to reach full empowerment at a community level. The programme can be seen as the first step of this process, but a lot of work remains to be done.

Gillies [9] stated that MSA health promotion collaborations between public, private and non-government sectors do work. Based on our findings, we agree. Nevertheless, we conclude that cooperation in a penta-helix structure is problematic in institutional contexts with hierarchical structures. Too many isolated downpipes in an organisation make it impossible to work in a governance manner. Cooperation in a penta-helix collaboration does work in a local context, however, provided you can work horizontally, in a governance system.

Arguably, policy changes for a penta-helix structure with citizen involvement in one area in the city of Malmö are not enough to reduce health inequalities within our society. It is therefore important to evaluate the implemented model in relation to health processes and outcomes among the citizens, in a way that also takes into account empowerment processes. Still, as stated by Freudenberg and Tsui [31], policy and practice changes reducing health inequalities do not occur naturally as a consequence of scientific evidence, the mobilization of a few communities, or the convictions of a few politicians. Instead, these changes result from multiple actions in many domains (ibid.).

This study demonstrates some methodological limitations as well as strengths. The qualitative analysis used in the study aims Participatory action research at searching for common patterns in the descriptions from a diverse sample, rather than comparing perspectives. The longitudinal design used for data gathering also allows for monitoring changes in reasoning over time amongst partners, which is especially important when implementing changes in organisational structures. The interview guide used [23] has been validated earlier and can also be used in future similar studies. However, regarding transferability, in this study we can only conclude that this partnership works in this context. It is a contextual study, with a narrow selection, having been conducted in a limited community. Abma et al. [10] have shown that evaluating local projects at different geographical sites can contribute to knowledge about working with CBPR models globally.

## Conclusions

The issue uncovered in this study is the problem of vertically organised institutions where discovery and innovation flow down from the top, thereby eliminating the essential input of the people and community that they are supposed to serve. This is a common challenge in health and social services and must be changed in order to improve and sustain health equity, healthcare and health outcomes. WHO states that an MSA, i.e., working with intersectoral actions, is important for reaching equality in health. The current programme strengthens the role of communities finding new models for this kind of collaboration.

The success of the programme is based on an interprofessional cooperation at a local level in a penta-helix structure where the citizens cooperate on equal terms. By working from a local context, close to the citizens, it is possible to give citizens an increased impact. Our findings indicate that local professionals working together with voluntary organisations and health promoters in a penta-helix structure on a local level could empower communities. We could also see the importance of strategic planning in a CBPR model to make the penta-helix structure work in practice. The programme made empowerment processes possible because of health promoters’ multidisciplinary work that did not have to align with existing structures.

For the future, more power and an increased mandate to decide must be given to the professionals on a local level to enable them to work in a penta-helix perspective. Citizens, such as the health promoters, must in the future also be established on a policy level and properly organised in the local context in sustainable ways. One solution, based on the findings from this study, is to work horizontally and solve problems at a local level based on citizens’ own needs. Future research studies should therefore also focus on an evaluation of such new organisational models and power issues in relation to health promotion among citizens. As a final note, since the interviews in this study were performed, the secretariat together with the health promoters have now converted into an association that continues the programme, and the research is organised by Malmö University. The steering group now works as an advisory board. These changes also support the sustainability of the penta-helix model and allow for future research.

## Data Availability

The interviews are not publicly available due copyright issues and GDPR regulations but are available from the corresponding author on a reasonable request.

## Abbreviations

CBPR: Community Based Participatory Research
MSA: Multisectoral approach
PAR: Participatory Action Research
WHO: World Health Organization

## References

[1] Marmot M (2005). Social determinants of health inequalities. Lancet.

[2] Wallerstein N (1992). Powerlessness, empowerment and health: implications for health promotion programs. Behav Chang.

[3] WHO. Multisectoral and intersectoral action for improved health and well-being for all: mapping of the WHO European Region. Governance for a sustainable future: improving health and well-being for all. Final Report. 2018. https://www.euro.who.int/__data/assets/pdf_file/0005/371435/multisectoral-report-h1720-eng.pdf. Accessed 23 Feb 2021.

[4] WHO. (2014). WHO | Health in All Policies: Framework for Country Action. https://www.who.int/cardiovascular_diseases/140120HPRHiAPFramework.pdf. (Accessed September 14, 2020).

[5] WHO. (2013). The Helsinki Statement on Health in All Policies, pp. i17–i18. https://www.who.int/healthpromotion/conferences/8gchp/8gchp_helsinki_statement.pdf (Accessed September 14, 2020).

[6] Jackson SF, Perkins F, Khandor E, Cordwell L, Hamann S, Buasai S (2006). Integrated health promotion strategies: a contribution to tackling current and future health challenges. Health Promot Int.

[7] Corbin JH, Mittelmark MB (2008). Partnership lessons from the global programme for health promotion effectiveness: a case study. Health Promot Int.

[8] Corbin JH, Jones J, Barry MM (2018). What makes intersectoral partnerships for health promotion work? A review of the international literature. Health Promotion Int.

[9] Gillies P (1998). Effectiveness of alliances and partnerships for health. Health Promot Int.

[10] Abma TA, Cook T, Rämgård M, Kleba E, Harris J, Wallerstein N (2017). Social impact of participatory health research: collaborative non-linear processes of knowledge mobilization. Educ Action Res.

[11] Holkup PA, Tripp-Reimer T, Salois EM, Weinert S (2004). Community-based participatory research: an approach to intervention research with a native American community. Adv Nurse Sci.

[12] Wallerstein N, Duran B, Oetzel J, Minkler M (2018). Community based participatory research for health: advancing social and health equity.

[13] Lewin K (1948). Resolving social conflicts.

[14] Freire P (1970). Pedagogy of the oppressed.

[15] Lindacher V, Curbach J, Warrelmann B, Bransetter S, Loss J (2017). Evaluation of empowerment in health promotion interventions a systematic review. Eval Health Prof.

[16] Belone L, Lucero JE, Duran B, Tafoya G, Baker EA, Chan D, Chang C, Greene-Moton E, Kelley MA, Wallerstein N (2016). Community-based participatory research conceptual model: community partner consultation and face validity. Qual Health Res.

[17] Ramji R, Carlson E, Brogårdh-Roth S, Nilvéus Olofsson A, Kottorp A, Rämgård M (2020). Understanding behavioural changes through community-based participatory research to promote oral health in socially disadvantaged neighbourhoods in southern Sweden. BMJ Open.

[18] Malmö stad. (2019). https://malmo.se/Fakta-och-statistik/Statistik-for-Malmos-omraden.html. (Accessed September 14, 2020).

[19] Israel BA, Schutz AJ, Parker EA, Becker AB (1998). Review of community-based research: assessing partnership approaches to improve public health. Annu Rev Public Health.

[20] Axelsson R, Bahari Axelsson S (2009). From territoriality to altruism in interprofessional collaboration and leadership. J Interprof Care.

[21] Ramji R, Carlson E, Kottorp A, Shleey S, Awad E, Rämgård M (2020). Development and evaluation of physical activity intervention informed by participatory research – a feasibility study. BMC Public Health.

[22] Renblad K, Henning C, Jegermalm M. Future Workshop as a Method for Societally Motivated Research and Social Planning. In: Henning C, Renblad K. Perspectives on empowerment, social cohesion and democracy: an international anthology. Jönköping: School of Health Sciences, Jönköping University; 2009.

[23] Wallerstein, N. (2017). Partnership Interview Guide; Qualitative Study Instrument, 2011 (adapted 2015), University of New Mexico Center for Participatory Research. From ‘Research for Improved Health: A National Study of Community-Academic Partnerships’ (2009–2013). Interview Guide for Self-Reflection and Evaluation of CBPR Partnerships: Version 2017. Adapted from Research for Improved Health: A study of Community-Academic Partnerships. https://cpr.unm.edu/research-projects/cbpr-project/index.html (Accessed September 14, 2020).

[24] Elo S, Kyngäs H (2008). The qualitative content analysis process. J Adv Nurs.

[25] Ortiz LM (2003). Multicultural health brokering: bridging cultures to achieve equity of access to health (Doctoral dissertation, University of Alberta).

[26] Svanholm S, Carleby H, Viitasara E (2020). Collaboration in health promotion for newly arrived migrants in Sweden. PLoS One.

[27] Mendes R, Plaza V, Wallerstein N (2014). Sustainability and power in health promotion: community-based participatory research in a reproductive health policy case study in New Mexico. Global Health Promot.

[28] Bodkin A, Hakimi S (2020). Sustainable by design: a systematic review of factors for health promotion program sustainability. BMC Public Health.

[29] Flynn BC, Wiles Ray D, Rider MS (1994). Empowering communities: action research through healthy cities. Health Educ Q.

[30] Laverack G. Health promotion practice: power and empowerment. London: SAGE Publications Ltd; 2004.

[31] Freudenberg N, Tsui E (2014). Evidence, power, and policy change in community-based participatory research. Am J Public Health.

